# Geographical and Climatic Relations of Pneumonia

**Published:** 1882-09

**Authors:** 


In studying the Geographical and Climatic relations of Pneumonia Dr. E. Sanders has summarized the following conclusions. His studies were based upon statistics obtained from many towns and cities in the United States and Europe:

"1st. The relations of pneumonia to altitude are definite and marked; with increase in elevation above the level of the sea, there is a steady diminution in the death-rate of pneumonia. To this rule some exceptions exist, but in the large majority of instances the relation holds good.

2d. The mean annual rainfall of a place bears no positive relation to pneumonia; in some instances a large mortality from the disease coincides with a large precipitation of rain, in others with a small precipitation, while in many others the contrary conditions are found to prevail.

3d. The higher the death-rate of a place from all causes, the greater the mortality from pneumonia. This rule is almost, if not actually, absolute.

4th. The larger the actual population of a locality, the greater its relative death-rate from pneumonia; the explanation for this being probably found in the following: Density of population bears an undoubted relation to the pneumonia rate, increase in the former being followed by, or going hand in hand with, increase in the latter.

5th. There is a direct, positive and unequivocal relation between the mean annual temperature of a place and its death-rate from pneumonia; the rule being that a high mortality from the disease coincides with a high mean temperature. Exceptions exist, but being unusual and rather rare, their existence can hardly be considered to invalidate this rule.

6th. Proximity to large bodies of water, such as lakes, inland seas, or the ocean, exerts no appreciable influence on the pneumonia rate.

7th. For North America, pneumonia increases in frequency as we pass from east to west; for Europe as we advance from west to east, the rate of increase being nearly twice as great in the case of the latter as in that of the former.

8th. Pneumonia, all other things being equal, increases in frequency the further we advance from the polar regions towards the tropics; this, however, only up to a certain parallel, beyond which it seems to become less and less commonly met with, until at or near the equator, when it apparently disappears. As far as the latter part of this statement is concerned, such would seem to be the truth, judging by what few facts are at hand. Statistics for the equatorial regions are rare and, even then, unreliable; hence I have purposely omited to present them. So few, vague and indefinite are they as to be almost valueless, allowing only of problematic deductions."—*American Journal of Medical Sciences*, July, 1882.

---

APHORISMS IN OPHTHALMOLOGY.—At the annual meeting of the Medico-Chirurgical Society of Maryland, Dr. J. J. Chisolm treated of eserine, which has taken the place of all other remedies in corneal affections; pilocarpin, which had produced the best results in the author's hands, internally used, of all the articles in the pharmacopeia in acute inflammations of the eyeball; salicylate of sodium, of the greatest value in iritic and scleral inflammations, whether specific or rheumatic; homatropin, to be preferred to atropia and duboisia on account of its transient mydriatic effect.

He also laid down the following aphorisms for general practitioners practicing ophthalmology:

1. Do not blister. 2. Do not use nitrate of silver. 3. Do not use acetate of lead. 4. Treat affections of the mucous surface by weak astringents—borax, sulph. zinc, tannin—and cleanliness. 5. Use weak solutions of eserine for corneal affections. 6. Atropia solutions are essential to break up recent iritic adhesions. 7. When in doubt, consult early with a specialist.—*The American Practitioner.*

